# Swallowing assessment in obstructive sleep apnea: insights from surface electromyography

**DOI:** 10.1007/s11325-026-03695-y

**Published:** 2026-05-02

**Authors:** Gislaine Aparecida Folha, Denny Marcos Garcia, Cláudia Maria de Felício

**Affiliations:** 1 Department of Health Sciences, Ribeirão Preto Medical School, University of São Paulo, 120 Miguel Covian Ave., USP Campus, Ribeirão Preto, São Paulo Brazil; 2 Craniofacial Research Support Center, University of São Paulo, Ribeirão Preto, São Paulo Brazil; 3 Department of Otorhinolaryngology, Ophthalmology, and Head and Neck Surgery, Ribeirão Preto Medical School, University of São Paulo, Ribeirão Preto, São Paulo Brazil

**Keywords:** Obstructive sleep apnea, Deglutition, Deglutition disorders, Surface electromyography

## Abstract

**Purpose:**

To investigate the electromyographic activity (sEMG) of the suprahyoid/submental (SH) muscles during swallowing in adults with different OSA severities, controlled for body mass index (BMI), and to establish predictive factors for changes in muscle activity.

**Methods:**

This cross-sectional observational study included 37 adults diagnosed with OSA (AHI > 5). The patients were divided into two groups according to the apnea-hypopnea index (AHI): Group I (AHI = 5 ≤ 30) and Group II (AHI > 30). sEMG activity was recorded during the voluntary swallowing of 10 mL and 15 mL of thin liquid (water). The sEMG peak, integral, and maximum velocity (Vmax) were calculated. Groups were compared by general multivariate analysis of covariance, with BMI as a covariate. A general multivariate linear regression model (GRM) was applied to analyze the contribution of predictors to the EMG parameters. The significance level was set at *p* < 0.05.

**Results:**

There was a predominance of males (*n* = 24, 64.9%) and obese individuals (BMI > 30, *n* = 25, 67.6%) in the sample. Compared with Group I, Group II presented significantly lower peak, Vmax and integral values (P *≤* 0.006). The GRM revealed that the peak and integral were explained by the AHI and BMI, whereas the tongue volume and behavior during swallowing together explained the Vmax.

**Conclusion:**

Activation of the suprahyoid (SH) muscles was reduced in patients with severe OSA. In addition to disease severity, BMI and the myofunctional condition of the tongue also contribute to impaired activation. Taken together, these findings indicate that muscle weakness and deficits in motor control compromise the activation of SH muscles during swallowing.

## Introduction

 Breathing and swallowing are vital and closely related functions that require complex neuromuscular coordination for optimal performance and safety because the upper airway (UA) is a complex muscular tube shared by both the respiratory and digestive systems [1]. During inspiration, dilation of the UA allows unobstructed airflow into the lungs. In contrast, swallowing involves oropharyngeal compression to propel the food bolus through the pharynx and upper esophageal sphincter [1]. During bolus transit, swallowing also plays a critical role in protecting the UA. This is achieved by interrupting respiration (swallowing apnea), closing the epiglottis, and by the elevation and anterior displacement of the hyolaryngeal complex through tongue‒palate contact and contraction of the submental/suprahyoid (SH) muscles [2–3]. Additionally, the clearance of the pharynx and esophagus by swallowing is essential for protecting the airways and lungs from aspiration [4].

Respiratory disorders such as obstructive sleep apnea (OSA) affect swallowing [5], as repeated apneic episodes can result in hypoxia (reduced oxygen levels in the blood), hypercapnia (elevated carbon dioxide levels), and neuromuscular changes in the involved tissues, particularly in the pharyngeal region [6]. Swallowing dysfunctions can also impair breathing, increasing the risk of penetration and aspiration, that is, the entry of material into the airway [7], with pneumonia as a possible consequence.

The combination of anatomical and nonanatomical factors, such as a narrow pharynx, increased UA length, and reduced UA muscle activity (including the SH muscles) during sleep, contributes to the manifestation of OSA in adulthood [8–9]. Thus, since OSA is a chronic sleep-related upper respiratory condition [10]; characterized by recurrent collapse of the UA, it can result in intermittent hypoxia, sleep fragmentation, and overall health impairments [11]. Although many aspiration events described in OSA are silent or trace, their clinical relevance should not be underestimated. Recurrent microaspiration is a recognized risk factor for chronic airway inflammation, aspiration pneumonia, exacerbation of pulmonary disease, and reduced quality of life. Preventing aspiration through early identification of swallowing dysfunction may reduce respiratory complications, decrease healthcare utilization, and enhance overall functional outcomes [12].

In light of this, the assessment of swallowing in this population becomes essential for guiding appropriate therapeutic interventions and preventing complications. Previous studies have reported impairments in the sensorimotor patterns and swallowing function of patients with OSA [5, 10, 12]. Moreover, dysfunctions occurred regardless of OSA severity and facial profile, with a greater predisposition to inferior hyoid positioning and associated myofunctional and swallowing alterations [13].

Although imaging methods such as videofluoroscopic swallowing studies (VFSSs) are considered the “gold standard” for dynamic swallowing evaluation, VFSSs present several limitations. These include high cost, exposure to ionizing radiation, the requirement for a fixed body position during the exam, interpretative variability, a restricted observation window, the need for barium contrast, and logistical issues such as the need for specialized physical space.

For these reasons, and owing to the possibility of analyzing additional parameters, studies focusing on the relationship between breathing and swallowing have included the analysis of surface electromyography (sEMG), a noninvasive and safe method for evaluating muscle activity [3]. Typically, sEMG is performed in combination with other methods either during sleep [4, 14–15] or with awake participants [16–17].

According to the literature, during sleep, swallowing frequency decreases and becomes lower as sleep depth increases [4, 15]. Swallowing frequency during sleep is greater in individuals with OSA [4, 14] and is especially associated with respiratory event-related arousals in patients with severe OSA than in those with mild OSA [14]. With respect to the swallowing‒breathing pattern, it is generally accepted that healthy adults exhibit swallowing followed by expiration, whereas in patients with OSA, most swallows during sleep are followed by inspiration [4], suggesting an out‒of‒phase pattern [15] and increasing the risk of aspiration.

In addition to information about the timing of events addressed in the cited studies, sEMG provides parameters related to muscle activation during swallowing, which have been explored in only a few studies involving patients with OSA [16–17]. However, sEMG of the suprahyoid muscle region is recommended for investigating swallowing dysfunctions, including the early detection of dysphagia, because of its role in bolus propulsion and airway protection [2–3].

Advancing in the study of the relationship between OSA and potential impairments in orofacial sensorimotor function has the potential to contribute to a better comprehension of the underlying anatomophysiological mechanisms of OSA and may influence therapeutic approaches.

Therefore, the objective of this study was to analyze suprahyoid muscle activation during swallowing in patients with different severities of OSA by means of sEMG. The associations of sEMG measurements with sex, age, BMI and the clinical assessment of tongue myofunctional status were also investigated.

## Methods

All procedures performed in studies involving human participants were in accordance with the ethical standards of the Research Ethics Committee of the University Hospital (protocol number 12634/2010) and with the 1964 Helsinki declaration and its later amendments or comparable ethical standards. Informed consent was obtained from all individual participants included in the study.

### Sample selection

The sample for this study consisted of consecutive patients who were diagnosed with OSA without prior treatment and were recruited at a university hospital.

#### Inclusion and exclusion criteria

Participants were eligible for inclusion in the OSA group if they presented clinical symptoms suggestive of OSA and had a confirmed diagnosis by overnight polysomnography (PSG), with an apnea-hypopnea index (AHI) greater than 5.0 events per hour of sleep. OSA diagnosis was established by overnight polysomnography according to the American Academy of Sleep Medicine (AASM) International Classification of Sleep Disorders [18]. Additional inclusion criteria were age between 18 and 60 years and the presence of at least 28 permanent teeth.

The exclusion criteria included the presence of crossbite, periodontal disease, temporomandibular disorder, a history of central or peripheral neurological disorders, craniofacial malformations, a history of head and neck surgeries, tumors, or trauma in the region. Individuals who had undergone orthodontic treatment, physiotherapy, or speech-language therapy within the two years prior to the study were also excluded. Other exclusion factors were chronic use of analgesics, anti-inflammatory drugs, or psychotropic medications, as well as pregnancy.

## Clinical examination and diagnosis

### Procedures

The study participants were individually instructed about the assessments, had the opportunity to practice the tests before the recordings, and remained seated in a comfortable chair with back support and with their feet on the floor. All procedures were performed in a quiet and private room at the Stomatognathic System Investigation Laboratory.

### sEMG analysis

sEMG activity was recorded via a wireless electromyographic system (FreeEMG, BTS S.p.A., Garbagnate Milanese, Italy). The analog sEMG signal was amplified and digitized (16-bit resolution, sensitivity of 1 µV, temporal resolution of 1 ms–1 kHz) via a differential amplifier with a high common mode rejection ratio (CMRR > 110 dB, in the range of 50–60 Hz, input impedance > 10 GΩ) [3]. Custom software developed in the MATLAB R2015a environment (version 8.5, MathWorks Inc., Natick, MA, USA) was used for data analysis. All recordings were digitally filtered via a second-order Butterworth bandpass filter with cutoff frequencies of 20–450 Hz. Next, the EMG signal was rectified, and a linear envelope was created via a low-pass Butterworth filter with a cutoff frequency of 5 Hz [3, 19].

The SH muscles were detected by palpation after the subjects swallowed saliva with commands, and the electrodes were bilaterally positioned in the submental region below the chin over the muscle mass felt during contraction [3]. Light tubes (11.7 g) were fixed to pregelled bipolar Ag/AgCl disposable electrodes (sensor area, 3.14 cm²; interelectrode distance, 2 cm; Kendall, Covidien, Mansfield, Canada). Before electrode placement, the skin was cleaned with 70% alcohol to reduce impedance. Images were recorded with three video cameras (frontal and both sides) simultaneously with the EMG to aid in confirming the duration (time) of hyolaryngeal complex elevation during analysis.

#### Swallowing tasks

Thin liquid (International Dysphagia Diet Standardized Initiative 0) was swallowed (voluntary): a cup containing water at a temperature of 26 to 28 °C was offered to the participants. They were instructed to swallow the entire content at one time after the examiner’s command. The test was performed separately with two volumes: 10 and 15 mL.

These tasks were selected to allow the participants to swallow only once and because they occur many times throughout the day. Two repetitions have been performed by all participants. During the tasks, the sEMG activity of the SH muscles was recorded.

After the sEMG signal was processed, the mean values for the right and left muscle pairs were determined. Because the swallowing variables were calculated within each interval of onset and offset, in each task, a mean basal reference value [average value of the EMG signal (filter/envelope) within a range, indicating a period in which there is no muscle activity related to swallowing or other movement] plus 3 standard deviations (*R* + 3*SD) was used to detect the onset and offset positions of SH muscle activity [3, 19].

The following variables were calculated automatically:

- Peak (µV): maximum value of the sEMG signal;
- Integral (µVs): area under the sEMG signal;
- Maximum velocity (Vmax, µV/s): maximum velocity of the sEMG signal until the peak was reached.

### Tongue evaluation

Tongue volume and tongue behavior during swallowing were assessed via the protocol for orofacial myofunctional evaluation with scores expanded (OMES-E), which was validated for patients with OSA according to a previously described methodology [20]. The evaluation was performed via visual inspection during the session and later confirmed via analysis of video-recorded images. A predetermined 4-point scale, with scores ranging from 1 (severe alteration) to 4 (normal), was used.

Tongue volume was classified as compatible with the oral cavity (normal, score of 4) or increased in volume and/or an enlarged tongue of mild (score of 3), moderate (score of 2) or severe grade (score of 1).

To evaluate tongue behavior during the swallowing evaluation, the subjects were asked to swallow as usual. The examiner explained that the subject’s lips would be separated immediately after swallowing (with the examiner placing the index finger and thumb, respectively, under the subject’s chin and lower lip (region of the mentalis muscle)). It was reinforced that this would not happen while the subject had water in his mouth. This procedure was used to visualize his teeth or even his tongue in case of deviation from normal behavior.

Tongue behavior was rated as normal (score of 4) when it was contained in the oral cavity and was therefore not visible. Deviant tongue behaviors were rated as follows, according to the vertical dimension of occlusion (VDO): tongue placed in the limit incisal surfaces of the teeth (or margins, in the absence of incisors) with a reduced VDO (score of 3); tongue placed on the limits of the incisal surfaces with a normal VDO (score of 2); tongue placed beyond the incisal surfaces and/or the vestibular cusps (score of 1). When the participant presented malocclusion as an anterior open bite and abnormal overjet, the following scores were attributed according to tongue placement: in the limit incisal surfaces of the teeth (score of 3); beyond the incisal surfaces and/or vestibular cusps in a moderate manner (score of 2); and beyond the incisal surfaces in an excessive manner (score of 1).

Tongue volume and behavior during swallowing were summed for analysis. Thus, the maximum possible tongue combined score was 8, and the minimum score was 2.

A trained and reliable speech-language pathologist (GAF) performed the myofunctional evaluation and, together with a medical physicist (DMG), conducted the sEMG recordings and analyses. Both were blinded to the groups to which the participants were allocated.

### Sample size calculation

Previously obtained descriptive statistics [3, 19] were used to estimate the minimum number of participants required in sample for a statistical analysis with 80% statistical power (type II error, beta) and alpha (type I error) set at 5%. Considering that group I could have twice as many participants as group II, the sample size for Peak, Vmax and Integral was, 26 (17 + 9), 30 (20 + 10) and 24 (16 + 8), respectively.

### Statistical analysis

Calculations were made via Statistica software version 14.0015 (TIBCO^®^ Data Science, Palo Alto, CA, USA) and R version 4.1.1 (R Foundation for Statistical Computing, Vienna, Austria). The level of significance was set at 5%.

Descriptive statistical analysis was computed for all variables. Categorical variables are presented as frequencies and percentages. Continuous data are expressed as the means and standard deviations (SDs) or as least square means with standard errors and 95% confidence limits. The Kruskal‒Wallis test was used for nonnormally distributed data, whereas the chi‒square test was used for categorical data. sEMG data with a nonnormal distribution were log-transformed. A paired t test was used to verify possible differences between the mean sEMG signals of the swallows of 10 and 15 mL. |Because there was no significant difference, the mean values for 10 and 15 ml were calculated.

To investigate differences in the sEMG variables between the OSA groups, a multivariate analysis of covariance (general linear model) was conducted. Planned comparisons of least squares (LS) means were used for pairwise comparisons, controlling for the covariate. The effect size (ES), which potentially indicates the clinical relevance of the results regardless of the sample size, was obtained via generalized eta squared (𝜂2𝑝𝜂*p2*) (Cohen, 1988), and the effects were interpreted as small, moderate, and large, respectively: (𝜂2𝑝𝜂*p2*) = 0.0099, (𝜂2𝑝𝜂*p2*) = 0.0588, (𝜂2𝑝𝜂*p2*) = 0.1379. Logistic regression (for sex) and univariate linear regression (for age, BMI and AHI) were performed to verify the associations between predictors and dependent variables (sEMG parameters). *P* < 0.20 was used as the selection criterion for covariates. Moreover, the contributions of the predictors to the sEMG parameters were determined via a multivariable linear regression model (GRM). Two models of regression analysis were performed: the first model investigated the contribution of the AHI and BMI, and the second model included the tongue combined score.

The reliability of the swallowing sEMG parameters obtained in our laboratory was previously shown by the interclass correlation coefficient (ICC) and test and retest recordings (paired Student’s t test) [3]. Two repetitions has been performed by all participants. Baseline was defined as a mean basal reference value (uV) [average value of the EMG signal (filter/envelope) within a range, indicating a period in which there is no muscle activity related to swallowing or other movement]. This value plus 3 standard deviations (*R* + 3*SD) was used to detect the onset and offset positions of SH muscle activity [3, 19].

## Results

### Demographic and clinical characteristics

All selected participants (*n* = 37) had OSA and were divided into two groups according to the AHI: mild/moderate (AHI 5 to ≤ 30) and severe (> 30 events/hour of sleep); Group I and Group II, respectively.

In the sample, the number of males was greater than that of females (24 vs. 13), as was the number of obese participants (BMI > 30 kg/m²) (*n* = 25).

The proportions of overweight (BMI from 25.0 to 29.9 kg/m²) and obese individuals were respectively in Group I of 26% and 61%, and in Group II 14% and 78%.

Only 13% of the Group I participants and 7% of the Group II participants had BMIs ranging from 18.5 to 24.9 kg/m². The statistical tests revealed a greater proportion of males in Group II than in Group I. Group I had the highest median age, although the group comparisons did not reach statistical significance. BMI also did not differ between groups, but it was included in the analysis because of its relationship with sEMG parameters. The demographic and anthropometric characteristics of the groups are presented in Table 1.

### Univariate analysis

Logistic regression revealed no associations between sex and the EMG variables, with *P* values ranging from 0.34 to 0.46. In the univariate linear regression, the AHI, BMI and Tongue combined score were associated with the sEMG peak (*R* = 0.50, *P* = 0.001), (*R* = 0.53, *P* < 0.001) and (*R* = 0.50, *P* = 0.002), respectively, and with the Vmax (*R* = 0.38, *P* = 0.020), (*R* = 0.38, *P* = 0.021), (*R* = 0.55, *P* < 0.001) and Integral (*R* = 0.46, *P* = 0.005), (*R* = 0.53, *P* < 0.001) and (*R* = 0.28, *P* < 0.09), respectively. Age was not significantly associated with the sEMG peak (*R* = 0.002, *P* = 0.99), Vmax (*R* = 0.12, *P* = 0.50) or integral (*R* = 0.21, *P* = 0.203) values. Therefore, sex and age were not included in the following analyses.

**Table 1:** Demographic, anthropometric, and clinical characteristics of groups with OSA

	Group I (*n* = 23)		Group II (*n* = 14)		*P* value
Sex male *n* (%)	12 (52.2)		12 (85.7)		0.038^a^
	Mean (SD)	Median (IQR)	Mean (SD)	Median (IQR)	
Age (years)	47.3 ± 9.8	49 (41:55)	42.4 ± 10.9	44.5 (37:50)	0.16^b^
BMI (kg/m^2^)	31.4 ± 6.7	32 (26.9:34.2)	32.2 ± 4.9	33.3 (30.3:35.8)	0.23^b^
AHI	16.2 (7.3)	13.5 (11.2:23.3)	65.1 (24.9)	63.5 (42.2:89)	< 0.0001^b^
Tongue combined score	4.8 (1.2)	5.0 (4.0:5.0)	4.1 (1.6)	4.0 (3:5)	0.12^b^

*P*: probability in the statistical tests, ^a^Chi-square test, ^b^Mann‒Whitney test. *AHI* apnea‒hypopnea index, *BMI* body mass index, *SD* standard deviation, *IQR* interquartile range

### Multivariate analysis of covariance

Because BMI showed a significant relationship with each sEMG parameter (peak, VMax, and integral) in the univariate linear regression, it was included as a covariate. Both the Group factor (groups I vs. II) and BMI demonstrated significant associations with the sEMG-dependent variables. Overall, the effect size was large. The results are presented in Table 2.

**Table 2:** Multivariate analysis. Dependent variables: peak, Vmax and integral; categorical factors: groups; continuous predictor: BMI

(n=37)				
Factor and covariate	Wilks λ	F	*P*	Effect size 𝜂2𝑝𝜂p2
Group	0.54	9.3	0.0004	0.465
BMI	0.57	8.0	0.0001	0.427

*P* value for a multivariate analysis of covariance; significant: *P* < 0.05

Effect size: generalized eta squared

### Pairwise comparison of LS means

After controlling for covariates (BMI), Group II presented lower and significant LS means for Peak, Vmax, and Integral during swallowing (Table 3).

**Table 3:** Electromyographic variables during water swallowing. Pairwise comparisons of the least squares means of the groups

		Group I	Group II	*P*
Peak (µV)	Mean ± SEM	3.76 ± 0.06	3.29 ± 0.08	< 0.0001
	95% CI	3.64–3.88	3.14–3.44	
Vmax (µV/s)	Mean ± SEM	5.62 ± 0.09	5.09 ± 0.12	0.001
	95% CI	5.43–5.81	4.85–5.83	
Integral (µV*s)	Mean ± SEM	3.04 ± 0.07	2.70 ± 0.09	0.006
	95% CI	2.89–3.18	2.51–3.88	

The EMG data of the variables were log-transformed. Mean: LS mean (adjusted for BMI), *SEM *standard error of the mean, 95% *CI* confidence interval, 10 mL and 15 mL of water swallowing; *Vmax* maximum velocity

### Multivariate linear regression analysis

Multivariable linear regression analysis was conducted to determine the contribution of OSA severity, as measured by the AHI, together with other factors. Models 1 and 2 were significant for all three sEMG parameters. For both models, the AHI and BMI contributed negatively. Thus, the higher the AHI or BMI is, the lower the peak and integral. However, Model 2 was found to be the most appropriate model because only the tongue combined score significantly explained the variability in Vmax, in addition to contributing to peak variability. The better the tongue functionality is, the higher the Vmax and peak (Table 4).

**Table 4:** Multivariate linear regression analysis of potential predictors of changes in sEMG parameters

		Peak (µV)	Vmax (µV/s)	Integral (µV*s)
Whole model 1	*R* ^2^	0.43	0.23	0.40
	*P*	< 0.0001	0.01	< 0.001
AHI	ß	-0.40	-0.31	-0.34
	95% CI	-0.67: -0.12	-0.66: -0.01	-0.62: -0.06
	*P*	0.005	0.056	0.017
BMI	ß	-0.43	-0.30	-0.45
	95% CI	-0.71:-0.16	-0.66:-0.01	-0.73:-0.17
	*P*	0.007	0.060	0.001
Whole model 2	R^2^	0.48	0.38	0.40
	*P*	< 0.0001	0.001	< 0.001
AHI	ß	-0.36	-0.24	-0.25
	95% CI	-0.63:-0.09	-0.01:0.0	-0.01:-0.001
	*P*	0.01	0.10	0.02
BMI	ß	-0.32	-0.10	-0.47
	95% CI	-0.62:-0.02	-0.43:0.22	-0.06: -0.01
	*P*	0.03	0.53	0.006
Tongue Cs	ß	0.25	0.44	-0.04
	95% CI	-0.05:0.55	0.12:0.77	-0.12:-0.91
	*P*	0.10	0.009	0.80

*AHI *Apnea‒Hypopnea Index. Tongue *Cs *Combined score of tongue volume and tongue behavior during swallowing

## Discussion

The present study enabled the identification of impairments in SH muscle activation during swallowing, as well as the contributing factors. The results revealed reduced activation of SH muscles in individuals with severe OSA, with lower Peak, Vmax, and Integral values than in those with an AHI < 30. Although BMI was not significantly different between groups, it was initially controlled to isolate the effect of OSA severity on sEMG parameters.

These findings add information, on the basis of a noninvasive methodology, regarding oropharyngeal swallowing impairments in patients with OSA, which are often underestimated and not yet fully understood [10, 12].

Insufficient function of the SH muscles during swallowing may impair tongue-to-palate coupling, as well as the elevation of the hyoid bone and larynx, thereby potentially compromising both bolus propulsion efficiency and upper airway protection [1]. In the present sample, no signs suggestive of impaired airway safety, such as coughing, throat clearing, choking, or wet voice, were observed during the ingestion of small volumes of water. This finding appears to confirm previous findings of subclinical dysphagia in patients with OSA, as identified through fiberoptic endoscopic evaluation of swallowing (FEES) [14, 21–22], VFSS, and pharyngeal manometry [10], which may worsen with advancing age [22].

The SH muscles, in addition to facilitating anterosuperior movement of the hyolaryngeal complex, contribute to pharyngeal dilation and stiffening (particularly the geniohyoid muscle), as well as to opening of the upper esophageal sphincter (UES) [20]. Recently, altered swallowing biomechanics, including increased UES relaxation pressure and a reduced UES opening diameter, have been identified in individuals with moderate to severe OSA [10].

In addition to its implications for swallowing, reduced activation of the SH muscles may lead to a lower hyoid position, which has been observed in patients with OSA and is associated with an increased likelihood of UA collapsibility [13, 23].

According to the results, worsening OSA was associated with reduced mean sEMG parameters, as the effect of BMI was “removed,” that is, statistically controlled in the group comparisons. Similar findings have been reported in children with OSA, who have lower peak and VMax values than healthy controls do [19], suggesting that such alterations may manifest as early as childhood. Sensory and motor deficits may help explain these results [12, 24]. One possible underlying mechanism is that nocturnal mechanical trauma caused by snoring and pharyngeal collapse in OSA patients may damage peripheral afferent nerves of the upper airway, impairing local sensitivity (e.g., the oral cavity and pharynx) and disrupting bolus detection and response mechanisms, thereby increasing the risk of swallowing dysfunction and even aspiration [12, 21]. However, in children, the impairment of sEMG variables, as well as tongue strength and reduced myofunctional condition scores, has been attributed to muscular weakness potentially associated with the number, diameter, and type of muscle fibers and to a deficit in sensory‒motor processing, compromising the stability and execution of vital functions such as swallowing and breathing during sleep [19].

The sample was predominantly male, which was expected given that participants were consecutive patients, aligning with the known male predisposition to OSA [10, 16]. However, neither sex nor age was included in the analyses because of the absence of associations with the EMG measures Peak, Integral, or VMax. According to the literature, no sex-related differences have been found in sEMG measurements of the SH muscles [2] or in the recruitment of motor units of the suprahyoid muscles assessed via high-density sEMG [25]. With respect to age, only older individuals have decreased EMG values [26]. Importantly, in the present sample, the maximum age was 59 years, with no difference between groups I and II.

The results of the multivariable linear regression analysis revealed that both increased AHI and higher BMI were associated with reduced SH muscle function, particularly in peak activity and integral values. Moreover, the myofunctional condition of the tongue, represented by the combined score, influenced Vmax (Model 2).

As previously described, obesity leads to an increase in upper airway soft tissues, including the tongue, and reduces the airway space. In turn, increased tongue size may displace the hyoid bone inferiorly and cause an increase in pharyngeal length [23]. Airway size is also regulated by the activation of upper airway muscles and can be reduced if muscle function is impaired. All these factors are interconnected and promote upper airway collapsibility [12].

The combined tongue score was designed to encompass not only volume—which, as previously discussed, is related to the airway space and collapsibility—but also its observable behavior during swallowing (function). As mentioned, the SH muscles contribute to tongue elevation against the palate and, together with the intrinsic and extrinsic tongue muscles, propel the bolus toward the pharynx [1]. The relative contribution of the tongue myofunctional condition accounted for 44% of the variance in VMax and 25% in peak, although the latter was not statistically significant. Therefore, patients with increased tongue volume (relative to the oral cavity space) and improper tongue positioning during swallowing tend to have lower peak activity but especially reduced VMax.

The finding that the combined tongue score was a significant predictor of Vmax, but not of the sEMG signal integral, suggests that tongue myofunctional condition is more closely related to the temporal dynamics and coordination of supra-hyoid muscle activation than to the total amount of sustained muscle activity during swallowing. Vmax reflects the rate at which muscle activation reaches its peak and is therefore sensitive to motor planning, neural drive, and the efficiency of tongue–palate coupling at the onset of swallowing. In contrast, the integral represents the accumulated electrical activity over time and may be more strongly influenced by swallowing duration, bolus transit time, and compensatory strategies than by the quality of motor coordination itself. Thus, alterations in tongue posture and function seem to primarily affect the speed and coordination of supra-hyoid muscle recruitment, captured by Vmax, rather than the total work performed during swallowing, reflected by the integral.

This interpretation has clinical utility and is supported by recent evidence from a randomized controlled trial [27], indicating that, in the short term, orofacial myofunctional therapy may promote stabilization of the hyoid bone position without immediate changes in global sleep-related outcomes, suggesting that structural and neuromuscular adaptations of the tongue–hyoid complex may precede measurable changes in cumulative functional measures. In this context, parameters sensitive to the dynamics of muscle activation, such as Vmax, may detect early changes in motor control, whereas integrative measures, such as the sEMG integral, may reflect later or compensatory adaptations.

However, it should be noted that although the regression models were statistically significant, some presented relatively low R² values, indicating limited explanatory power. Therefore, the observed associations should be interpreted as trends rather than definitive causal relationships. The contributions of BMI and the combined tongue score reflect their potential role in modulating suprahyoid muscle activation but do not imply causality.

Although no clinical signs of aspiration were observed, these findings highlight the potential relevance of incorporating myofunctional assessment of the tongue and suprahyoid region into the evaluation of patients with moderate to severe OSA, as reduced suprahyoid muscle activation during swallowing may compromise hyolaryngeal elevation and mechanisms related to airway protection [13].

Myofunctional exercises focusing on tongue volume and enhancing tongue-to-palate coupling during swallowing, along with other exercises aimed at improving dysfunctional upper airway muscles, have been shown to reduce OSA severity and symptoms in adults [28]. These factors could be investigated for their effects on SH muscles in patients with OSA. A study conducted with healthy volunteers concluded that effortful swallowing, emphasizing tongue‒palate contact, generates higher-velocity bolus driving forces that propel the bolus more efficiently through the pharynx, with sustained pressure facilitating more complete bolus clearance [29]. Recently, tongue strengthening training was shown to increase the sEMG activity of the SH muscles and improve perceived swallowing function in patients with Parkinson’s disease [30].

Swallowing is closely coordinated with respiration, and this coordination has been shown to be altered in individuals with OSA during sleep [4, 15]. Studies report increased latency to initiate swallowing, reduced inspiratory suppression time, and greater volume required to evoke the swallowing reflex [24]. These findings suggest not only structural alterations but also functional and sensory disturbances in swallowing control, affecting the integration of the respiratory and swallowing systems [5, 16]. This coordination is a fundamental mechanism for preventing laryngeal penetration and pulmonary aspiration [24].

Beyond orofacial myofunctional therapy, the observed association between BMI and suprahyoid muscle activation underscores the importance of weight management in patients with OSA. Interventions aimed at reducing body weight may indirectly influence upper airway anatomy and neuromuscular function. In addition, emerging therapies focusing on tongue endurance and neuromuscular control may be conceptually relevant to the present findings, as they target mechanisms related to motor coordination. In contrast, there is currently limited evidence that positive airway pressure, mandibular advancement devices or pharmacologic intervention that targets obesity including dual GIP/GLP-1 receptor agonists, directly improve tongue muscle strength or myofunctional behavior. These therapeutic perspectives should be interpreted as complementary and warrant further investigation. And, given the association between obesity, upper airway collapsibility, and neuromuscular function [23], future studies should investigate whether improvements in OSA severity translate into measurable changes in swallowing biomechanics after weight reduction.

The present study adds evidence that patients with severe OSA have reduced activation of the SH muscles during awake swallowing, reinforcing the presence of neuromuscular dysfunctions. It would be interesting to investigate whether such impairment translates into greater disruption of breathing-swallowing coordination during sleep, when loss of muscle tone is expected.

While sEMG of the suprahyoid muscle region should be regarded primarily as a research tool, the implementation of orofacial myofunctional assessment can offer valuable insights in clinical contexts, ultimately aiding in the understanding of neuromuscular factors related to OSA and tailoring treatment approaches, especially by validated instruments as the Orofacial Myofunctional Evaluation with Scores Expanded (OMES-E) [20]. Further studies are needed to explore the clinical utility of sEMG and to establish standardized indications for its routine use.

This study has some limitations. First, the sample size was relatively small, as the study was exploratory in nature, and prevented sex matching between participants in the groups. Second, individuals without OSA were not included, which limits the ability to determine how far the observed swallowing-related electromyographic patterns deviate from normal physiology. Future studies including healthy control groups and larger samples are warranted to confirm and expand these findings, and to investigate swallowing-related neuromuscular impairments across different OSA severity levels and subtypes. However, it is worth noting that previous studies by our research group have already conducted research using the same methodology in healthy individuals [3]. Furthermore, sEMG parameters were measured only with a thin liquid (water); tasks involving different volumes and consistencies could be useful in broadening the understanding of SH muscle function in patients with OSA.

## Conclusion

The group with greater OSA severity exhibited lower electromyographic activity of the suprahyoid muscles during liquid swallowing, suggesting muscle weakness and insufficient motor activation control of swallowing. BMI has emerged as a relevant modulator of muscle activity, reinforcing its role in therapeutic planning. However, the AHI remained significantly associated with reduced sEMG values. Additionally, clinical swallowing assessments, specifically the tongue combined score, were correlated with sEMG parameters in OSA patients.

These findings contribute to a deeper understanding of myofunctional dysfunction in OSA patients and underscore the need for therapeutic approaches that incorporate the functional rehabilitation of oropharyngeal muscles. 

## Data Availability

The authors have all the original data supporting the results and analyses presented in this study. Interested researchers may contact the authors directly to request access.
